# “Clicking” an Ionic Liquid to a Potent Antimicrobial Peptide: On the Route towards Improved Stability

**DOI:** 10.3390/ijms21176174

**Published:** 2020-08-26

**Authors:** Ana Gomes, Lucinda J. Bessa, Patrícia Correia, Iva Fernandes, Ricardo Ferraz, Paula Gameiro, Cátia Teixeira, Paula Gomes

**Affiliations:** 1 LAQV-REQUIMTE, Departamento de Química e Bioquímica, Faculdade de Ciências, Universidade do Porto, P-4169-007 Porto, Portugal; anagomes@fc.up.pt (A.G.); lucinda.bessa@fc.up.pt (L.J.B.); patricia.correia@fc.up.pt (P.C.); iva.fernandes@fc.up.pt (I.F.); ricardoferraz@eu.ipp.pt (R.F.); agsantos@fc.up.pt (P.G.); catia.teixeira@fc.up.pt (C.T.); 2 Ciências Químicas e das Biomoléculas, Escola Superior de Saúde, Politécnico do Porto, P-4200-072 Porto, Portugal

**Keywords:** antibacterial, antibiofilm, ionic liquid, multidrug resistance, peptide, skin infections, tyrosinase, wound healing

## Abstract

A covalent conjugate between an antibacterial ionic liquid and an antimicrobial peptide was produced via “click” chemistry, and found to retain the parent peptide’s activity against multidrug-resistant clinical isolates of Gram-negative bacteria, and antibiofilm action on a resistant clinical isolate of *Klebsiella pneumoniae*, while exhibiting much improved stability towards tyrosinase-mediated modifications. This unprecedented communication is a prelude for the promise held by ionic liquids -based approaches as tools to improve the action of bioactive peptides.

## 1. Introduction

Ionic Liquids (ILs), though mostly known for their potential role as “green solvents”, are becoming increasingly attractive as easily customizable and tunable organic salts for diverse specific purposes (task-specific ILs). There is an infinity of possibilities when combining organic cations with organic or inorganic anions, enabling production of ILs with diverse structural, physical and chemical properties [1] that can be adapted to the demands of areas as diverse as material sciences [2], biotechnology [3], or biomedicine [4]. Moreover, by making use of bioactive ions, ILs displaying relevant biological activities [5] can be produced, for instance, as anticancer [6], antimalarial [7], and antimicrobial agents [8].

ILs showing broad-spectrum activity against both Gram-negative and Gram-positive bacteria and antibiofilm activity have been reported [9]; as such, ILs are emerging as appealing alternatives to counteract antimicrobial resistance, while the world is running out of effective antibiotics, especially against Gram-negative bacteria. Moreover, many ILs have gained attention as dermal permeation enhancers [10], making them particularly attractive for topical applications. ILs presenting both antimicrobial activity and dermal permeation enhancement can be quite helpful to treat infected skin lesions, as recently demonstrated in an in vivo biofilm-infected wound assay, where an IL was able to kill 95% of the bacteria [11].

In view of the above, and based on our recent disclosure of a collagenesis-inducing peptide with potent antibacterial and antibiofilm properties [12], we now investigated the effect of coupling an antimicrobial methylimidazolium IL to the *N*-terminus of that peptide on antibacterial and antibiofilm activities, as well as on stability. To this end, we opted to use the well-known copper(I)-catalyzed alkyne-azide cycloaddition (CuAAC) “click” reaction, given its chemoselectivity and mildness, and the fact that it produces a stable triazole ring [13]. Moreover, CuAAC reactions have been previously used to introduce the triazole moiety in peptides aiming at, e.g., (i) replacing unstable bonds like disulfide bridges, (ii) improving peptide stability via cyclization, (iii) attaching distinct moieties and/or functional groups to the peptide, and (iv) taking advantage of the biological properties of many triazoles to improve overall bioactivity [14].

## 2. Results and Discussion

### 2.1. Chemical Synthesis

**Scheme 1:**
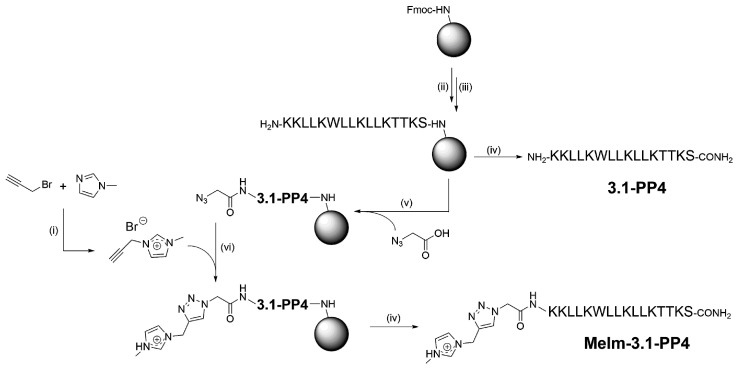
Synthesis route towards MeIm-3.1-PP4: (**i**) 1.1 molar equivalents (eq) of MeIm, 1.0 eq of propargyl bromide (80% in toluene), 40 ^°^C, 24 h; (**ii**) 5 eq of Fmoc-protected amino acid, 10 eq of *N*-ethyl-*N,N*-diisopropylamine (DIEA) and 5 eq of *O*-(benzotriazol-1-yl)-*N,N,N′,N*′-tetramethyluronium hexafluorophosphate (HBTU) in *N,N*-dimethylformamide (DMF), 1 h, room temperature (r.t.); (**iii**) 20% piperidine in DMF, 15 min, r.t.; (**iv**) trifluoroacetic acid (TFA)/triisopropylsilane (TIS)/distilled water (95:2.5:2.5 *v*/*v*/*v*), 2 h, r.t.; (**v**) 5 eq of azido acetic acid, 10 eq of DIEA, and 5 eq of HBTU in DMF, 1 h, r.t.; (**vi**) 1 eq sodium L- ascorbate, 10 eq of DIEA, 10 eq of 2,6-lutidine, 1 eq of Pr-MeIm, and 1 eq ofcopper(I) bromide in DMF:acetonitrile (MeCN) (3:1 *v*/*v*).

To produce the target IL-peptide conjugate, hereafter termed MeIm-3.1-PP4, the synthetic route shown in Scheme 1 was employed, starting with the synthesis of the alkyne-modified IL, by reacting 1-methylimidazole (MeIm) with propargyl bromide (step i), according to Hu et al. [15]; the structure of the target propargyl-MeIm (Pr-MeIm) was confirmed by proton (^1^H) and carbon (^13^C) nuclear magnetic resonance (NMR) and electrospray ionization-ion trap mass spectrometry (ESI-IT MS), as described in detail in the Supplementary Materials (Figures S1–S3). In parallel, the amino acid sequence of 3.1-PP4 was assembled by solid-phase peptide synthesis (SPPS, steps ii and iii) as previously reported by us [12]. Cleavage of half of the peptidyl-resin (step iv) afforded the parent peptide 3.1-PP4. The other half was coupled with azidoacetic acid (step v) to produce the azide-modified peptide; this was followed by on-resin CuAAC with Pr-MeIm (step vi), applying the conditions previously described by Castro et al. [16]. After cleavage and purification by reverse-phase high performance liquid chromatography (RP-HPLC), the target MeIm-3.1-PP4 conjugate was obtained in high purity (Figure S4), and its structure was confirmed by ESI-IT MS analysis (Figure S5). To the best of our knowledge, on-resin “clicking” of an ionic liquid to a peptide via the CuAAC has never been reported before.

### 2.2. Antibacterial and Antibiofilm Activity

Both the conjugate MeIm-3.1-PP4 and its parent peptide 3.1-PP4 were next screened for their antibacterial activity. Minimum inhibitory concentration (MIC) values were determined following the Clinical and Laboratory Standards Institute (CLSI) protocol [17], against Gram-positive (*Staphylococcus aureus* and *Enterococcus faecalis*), and Gram-negative (*Pseudomonas aeruginosa, Escherichia coli* and *Klebsiella pneumoniae*) bacteria, including both reference strains (American Type Culture Collection, ATCC), and multidrug-resistant (MDR) clinical isolates. Data thus obtained are shown in Table 1, and clearly demonstrate that, with only very few exceptions, attachment of the IL to the *N*-terminus of 3.1-PP4 did not significantly alter the antibacterial activity of the parent peptide. Relevantly, in most cases, MIC values matched the minimum bactericidal concentration (MBC) values, highlighting a bactericidal action for both MeIm-3.1-PP4 and its parent peptide.

**Figure 1:**
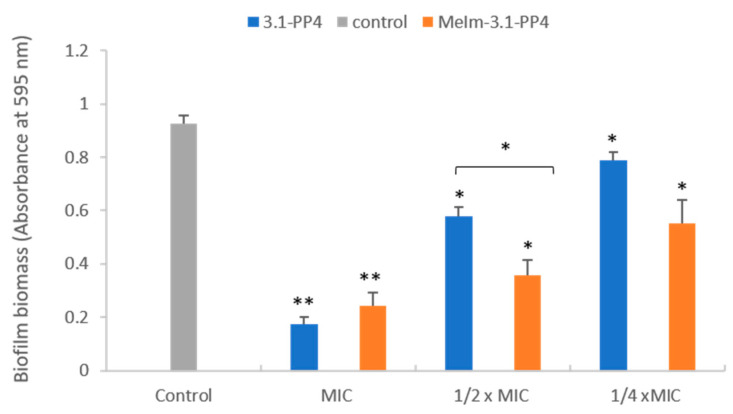
Effects of 3.1-PP4 and MeIm-3.1-PP4 on formation of *K. pneumoniae* KP010 biofilms (biomass quantification by crystal violet absorbance at 595 nm). Three independent experiments were performed in triplicate and the bar errors represent the SD. Statistically significant differences between the biofilm formed in absence and in presence of peptide are indicated: * means (*p* < 0.05) and ** means (*p* < 0.01).

Since this type of IL-AMP conjugate is being developed for its potential interest to address infected skin lesions, a preliminary assessment of the toxicity of both the conjugate and the parent peptide towards HaCaT human epidermal keratinocytes was carried out by the 3-(4,5-dimethylthiazol-2-yl)-2,5-diphenyltetrazolium bromide (MTT) assay, as previously described by us [12]. Despite the IC_50_ (24 h) obtained was somewhat lower for the conjugate (19.4 ± 0.2 µM) than for the parent peptide (34.2 ± 0.5 µM), the conjugate still exhibited selectivity indices ranging from ca. 14 to ca. 50 in most cases. In face of the excellent antibacterial properties exhibited by the MeIm-3.1-PP4 conjugate, especially against Gram-negative bacteria, we next evaluated its antibiofilm activity using the *K. pneumoniae* MDR isolate, KP010, since the parent peptide, 3.1-PP4, had previously displayed a more notable effect against this isolate [12]. This activity was assessed in two different ways: (i) inhibition of biofilm formation, and (ii) effect on a preformed biofilm. For evaluation of the inhibition of biofilm formation, both conjugate and parent peptide were tested at MIC and sub-inhibitory concentrations (½×MIC and at ¼×MIC) following previously established procedures [12,18,19,20], and the mass of biofilm formed at these concentrations was assessed through the crystal violet assay. Figure 1 shows results obtained in absence (control) and in presence of the test compounds at the three different concentrations. Relevantly, the absorbance measured for the control (absence of test compounds) provides confirmation that the bacterial strain used is a good biofilm producer [21]. As expected, only a little amount of biofilm was formed at MIC in both cases, with a slightly higher antibiofilm effect for the parent peptide. At sub-inhibitory concentrations (½×MIC and ¼×MIC), the biofilm biomasses formed remain lower than the control, in this case with a slightly stronger antibiofilm effect for the MeIm-3.1-PP4 conjugate.

To assess the effects on preformed biofilms, much higher peptide concentrations are needed, hence biofilms of KP010 were allowed to grow for 48 h and then treated with the peptides at 20× the MIC value for 24 h. Biofilm proliferation was determined by measuring the optical density (OD) of the planktonic phase of the biofilms, both treated with the test compounds and the untreated control. Results (Table S2 in Supplementary Materials) show that MeIm-3.1-PP4 and 3.1-PP4 caused a significant reduction in biofilm proliferation of 67% and 52%, respectively. Overall, these results show that the novel IL-peptide conjugate preserves not only the antibacterial but also the antibiofilm properties of the parent peptide. This improvement, afforded by the insertion of the imidazolium motif, is in line with previous reports of enhanced antibacterial activity when combining imidazolium ILs with non-peptide antibiotics, like ampicillin, including against resistant bacterial strains [22].

### 2.3. Enzymatic Stability

**Figure 2:**
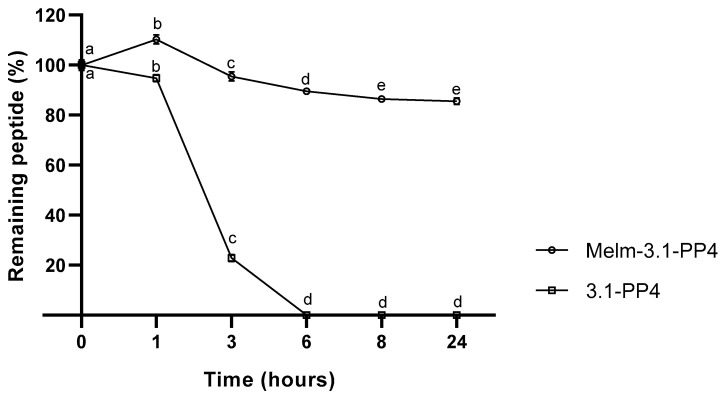
Compared stability of MeIm-3.1-PP4 and its parent peptide, upon incubation with tyrosinase (expressed as the variation of the % of starting compound over time) of three independent experiments (*n* = 3). Values are expressed as mean ± SD. Statistical significance, between the two test compounds for the same time point, was considered when *p* < 0.0001, and observed for all time points. Statistical significance, between time points for the same compound, was considered when *p* < 0.05 and is indicated by different letters.

One of our main motivations to explore IL-peptide conjugates concerns improving the enzymatic stability of the peptide moiety, as poor pharmacokinetics is a major obstacle towards clinical translation of AMP and other bioactive peptides, even when topical applications are envisaged. For instance, in a non-healing infected wound, peptides are vulnerable to modification by endogenous (e.g., tyrosinase, elastase, metalloproteinases) and exogenous (produced by colonizing microbes) enzymes in the wound site [23]. Peptide 3.1-PP4 and derivatives thereof are under investigation for topical applications on infected skin lesions; thus, to evaluate how the conjugation of an IL to 3.1-PP4 could affect the enzymatic stability of this peptide, we incubated both the conjugate and the parent peptide with tyrosinase, due to its central role in melanin biosynthesis in the skin [24], and reported increased levels in the course of wound healing [25]. The choice of this particular enzyme may seem odd at a first glance, as the 3.1-PP4 sequence lacks tyrosine residues, whereas tyrosinase is best known for promoting oxidation of phenols like, e.g., the side chain of tyrosine [25]. However, recent evidence of proteolytic activity for tyrosinase, with an apparent preference for hydrophobic amino acids, e.g., isoleucine, at the cleavage site [26], stimulated our interest in investigating whether our leucine-rich peptide would be a substrate for this enzyme of undeniable relevance in skin and wound healing. Hence, both parent peptide and its ionic liquid conjugate were incubated at 37 °C and pH 6.8 in the presence of the enzyme, and in its absence as a control. The samples were collected at different time points, for 24 h, and were analyzed by HPLC. Results are presented in Figure 2, where the complete degradation, within 6 h, of peptide 3.1-PP4 confirmed it as a substrate for tyrosinase, despite being devoid of tyrosine or any other phenol or polyphenol moieties. Additionally, about 84% of the IL-peptide conjugate MeIm-3.1-PP4 remained unaltered after 24 h, demonstrating the remarkable gain in stability afforded by conjugation of the IL to the *N*-terminus of the parent peptide. Both controls remained unchanged at the end of the experiment (data not shown).

The above findings are remarkable in two ways; (i) we confirm the recent observations by Biundo et al. in that tyrosinase can display promiscuous proteolytic activity [26], and (ii) we demonstrate that simple conjugation of an IL to the *N*-terminus of the peptide substrate prevents its degradation. Ongoing studies with these and other analogues will allow us to establish the pathway of peptide degradation by tyrosinase, and to ascertain how the peptide site at which the IL is conjugated (e.g., *C*-terminal rather than *N*-terminal conjugation) may influence enzymatic stability. Additional assays to evaluate conjugate’s stability in the presence of more common enzymes and enzyme mixtures (trypsin, pronase), as well as other enzymes that are relevant in the context of chronic wounds (collagenase, elastase, metalloproteinases), will be timely carried out.

## 3. Concluding Remarks

In conclusion, this exploratory work holds great promise for the future, by demonstrating that “clicking” a classical imidazolium IL to the *N*-terminus of a bioactive peptide can preserve its potent antibacterial and antibiofilm action, while significantly improving its stability towards an enzyme that is relevant in the skin wound environment. To the best of our knowledge, despite previous reports that have addressed both covalent [27] and non-covalent [28] combinations of IL with peptides, there is no precedent in using click chemistry to create IL-AMP conjugates, nor in demonstrating the substantial stabilization observed against tyrosinase-mediated modification upon conjugation of an IL to an AMP. Ongoing studies will allow us to build a wider landscape of enzymatic stability for MeIm-3.1-PP4 and similar IL-peptide conjugates that are being produced, by including other analogues, and other enzymes relevant in the context of infected skin lesions and wound healing. Moreover, hemolysis assays will be carried out in order to establish whether conjugation to the IL renders the final conjugate more hemolytic than the parent peptide, at the light of previous literature reports [27,29]. Furthermore, in vitro wound healing and dermal permeation assays are under way that will allow us to further demonstrate the expected high value of this unprecedented approach. Finally, the potent action of the compounds herein reported against Gram-negative bacteria is noteworthy, since despite peptide-based structures with potent action against Gram-positive bacteria are being disclosed [30,31,32,33], the situation is quite different regarding MDR Gram-negative bacteria, against which real alternatives to current antibiotics remain elusive.

## 4. Materials and Methods

### 4.1. Synthesis of 1-Methyl-3-Propargyl Imidazolium Bromide

To a round-bottom flask containing 1-methylimidazole (0.78 mL; 9.89 mmol), 80% propargyl bromide in toluene (1 mL; 8.99 mmol) was slowly added, and the deep yellow oily mixture formed was kept under stirring for 24 h at 40 °C. The mixture was then cooled down to r.t., and sequentially washed with dichloromethane (DCM, 10 mL) and diethyl ether (10 mL) [15]. The resulting yellow oil was dried in a vacuum oven overnight, at 50 °C and 0.1 bar, to afford the final product in 77% yield. ^1^H, ^13^C NMR and ESI-IT MS analyses allowed to confirm the product as being the desired 1-methyl-3-propargyl imidazolium bromide, according to spectral data below (spectra obtained are displayed in the Supplementary Materials—Figures S1–S3). NMR spectra were acquired on a Bruker Avance III400 spectrometer from solutions of the compound in hexadeuterated dimethyl sulfoxide (DMSO-d6), containing tetramethylsilane (TMS) as internal reference. Multiplicity of proton NMR signals is given as: s, singlet; d, doublet; t, triplet.

δ_H_ (400 MHz, DMSO-d6) 9.27 (s, 1H), 7.80 (t, 1H, J = 1.8 Hz), 7.76 (t, 1H, J = 1.8 Hz), 5.22 (d, 2H, J = 2.6 Hz), 3.88 (s, 3H), 3.84 (t, 1H, J = 2.6 Hz). δ_C_ (100 MHz, DMSO-d6) 136.3, 124.1, 122.1, 79.0, 76.1, 38.5, 36.0 (ESI+) ^m^/_z_ calculated for C_7_H_9_N_2_^+^, 121.08; found, 121.20.

### 4.2. Peptide Synthesis

The amino acid sequence of 3.1-PP4 (KKLLKWLLKLLKTTKS, *C*-terminal amide) was assembled by SPPS, using the standard Fmoc/^t^Bu orthogonal protection scheme [34]. Briefly, Fmoc-Rink-amide MBHA resin (100–200 mesh, 0.52 mmol/g) was swelled in DMF for 30 min, next treated with an excess of 20% piperidine in DMF for 15 min (r.t.); after several washing steps using DMF (3 × 10 mL) and DCM (3 × 10 mL), the Fmoc-protected *C*-terminal amino acid (5 eq) was coupled to the resin in the presence of the in situ coupling agent HBTU (5 eq) and the non-nucleophilic base DIEA (10 eq) in DMF, for 1 h at r.t., under stirring. After a few quick washing steps as before, the Fmoc group was removed with piperidine as above, and the coupling → deprotection cycle was repeated until the full amino acid sequence was assembled. At this stage, the peptidyl-resin was split in two equal portions, one of which was reserved for the subsequent stage (synthesis of MeIm-3.1-PP4, see Section 4.3). Peptide 3.1-PP4 was cleaved off the other portion of the peptidyl-resin via acidolytic cleavage with a cocktail containing 95% TFA, 2.5% TIS and 2.5% distilled water, for 2 h under stirring at r.t.. The crude peptide was then purified by preparative RP-HPLC, on a Hitachi-Merck LaPrep Sigma system using a C18 column (250 × 25 mm ID, 5 µm pore size). The elution gradient of 1 to 100% of MeCN, used 0.05% aqueous TFA as solvent A and MeCN as solvent B, and was run for 60 min at 15 mL/min. Fractions containing pure peptide were pooled, and an aliquot was taken to assess final purity degree by HPLC and confirm the expected molecular weight by ESI-IT MS, as previously described [17]. The final peptide solution was freeze-dried and stored at −20 °C until further use.

### 4.3. Synthesis of MeIm-3.1-PP4

To the reserved portion of peptidyl-resin having the 3.1-PP4 sequence fully assembled (see Section 4.2), a solution containing azidoacetic acid (18.7 mL; 5 eq), DIEA (85 mL; 10 eq), and HBTU (64 mg; 5 eq) in DMF was added, and the slurry kept under stirring for 1 h at r.t. After quick washing steps with DMF and DCM, as described in Section 4.2, a solution containing sodium L-ascorbate (10 mg; 1 eq), DIEA (85 mL; 10 eq), 2,6-lutidine (58 mL; 10 eq), and 1-methyl-3-propargyl imidazolium bromide (10 mg; 1 eq) in DMF (3 mL) was added, followed by addition of a solution of copper(I) bromide (7 mg; 1 eq) in MeCN (1 mL). The reaction was allowed to proceed at r.t. for 24 h, under stirring. Then, the resin was thoroughly washed with 1 M aqueous ethylenediaminetetraacetic acid (EDTA, 5 × 10 mL), followed by DMF and DCM as before. The crude product was cleaved from the resin via acidolysis, as in 4.2, and found (LC-MS) to be a mixture of the target conjugate (ca. 97%) and unreacted azido-peptide (ca. 2%), amongst other minor unidentified components, probably corresponding to truncated peptides (data not shown). The crude product was purified by preparative RP-HPLC, as done before for the parent 3.1-PP4 peptide (see Section 4.2). The pure conjugate was analyzed by HPLC (Figure S4) and ESI-IT MS (Figure S5), freeze-dried and stored at −20 °C until further use.

### 4.4. Peptide Quantitation

Peptide 3.1-PP4 and conjugate MeIm-3.1-PP4 stock solutions were prepared in distilled water at ca. 10 mg/mL. Concentration was then accurately determined on a Thermo Scientific™ NanoDrop™ One microvolume UV-Vis Spectrophotometer, using method 31 that assumes an extinction coefficient ε_205_ of 31 mLmg^−1^cm^−1^ and an A_280_/A_205_ correction for the absorbance due to tryptophan (Trp) and tyrosine (Tyr) residues eventually present (only Trp was present in this case) [35].

### 4.5. Antibacterial Activity Assays

The MIC of the test compounds was determined according to the CLSI guidelines [17]. Briefly, the compounds were tested in the 1–1024 µg/mL concentration range, using the microdilution method in cation-adjusted Mueller-Hinton broth (MHB II) against reference strains of *P. aeruginosa* ATCC 27853, *E. coli* ATCC 25922, *S. aureus* ATCC 25923, and *Enterococcus faecalis* ATCC 29212. MIC values were also determined against MDR clinical isolates (antibiotic resistance patterns given in Supplementary Materials, Table S1) of *P. aeruginosa* (PA004, Pa3, Pa4), *E. coli* (Ec2, EC001, EC002, EC003), *K. pneumoniae* (KP004, KP010), *S. aureus* (SA007), and *Enterococcus faecalis* (Ef1). Along with the MIC values, MBC values were also determined as previously reported [12].

### 4.6. Toxicity to Human Keratinocytes

The cytotoxicity of peptides to HaCat cells was evaluated using the standard MTT assay. Briefly, cells were seeded at a density of 4 × 10^4^ cells/mL, respectively, in 96-well plates and incubated at 37 °C in a 5% CO_2_ atmosphere. Cells were allowed to grow for 24 h, and serially diluted compound solutions (6.3–100 µM) were added to the wells. Then, cells were incubated for 24 h at 37 °C, after which wells were washed once with phosphate buffered saline (PBS, Sigma-Aldrich), followed by addition of a 0.45 mg/mL MTT solution to each well. Crystals were allowed to form for 1.5 h. Reaction was stopped by rejecting the medium followed by addition of dimethylsulfoxide (DMSO, Sigma-Aldrich). Absorbance was read at 570 nm in FlexStation^®^3 Multi-Mode Microplate Reader (Molecular Devices, San Jose, CA, USA).

### 4.7. Antibiofilm Activity Assays

The ability of the test compounds to inhibit the biofilm formation by KP010 (a MDR clinical isolate of *K. pneumoniae*) was assessed at the respective MIC, ^1^/_2_×MIC and ^1^/_4_×MIC values in tryptic soy broth (TSB) using the crystal violet assay as previously reported [36,37]. Three independent experiments were performed in triplicate for each condition.

To assess the effects of test compounds on 48 h preformed biofilms of KP010, the biofilms were first grown in TSB from a starting inoculum of 1 × 10^6^ CFU/mL in 96-well microtiter plates. After 48 h of incubation at 37 °C, the planktonic cells were removed, and the wells were rinsed and filled with the test compound at a 20× MIC concentration. The optical density at 600 nm was measured at time 0 and after incubation for 24 h at 37 °C. The reduction in the biofilm proliferation was calculated in comparison to the respective non-treated biofilms. Three independent experiments were performed in triplicate.

### 4.8. Enzymatic Stability Assays

The IL-peptide conjugate and the parent peptide were both incubated at a final concentration of 10 mM in phosphate buffer (pH 6.8), with the enzyme tyrosinase (CAS:9002-10-2; T3824-250KU) at 18 U/mL, for 24 h at 37 °C. Then, 150-μL samples were collected after 0, 1, 3, 6, 8 and 24 h of incubation, and 150 μL of methanol were added to each of them to precipitate the enzyme. The mixtures were centrifuged at 1.05 × 10^4^ RPM for 2 min with an Eppendorf miniSpin centrifuge (rotor F-45-12-11). The supernatant was then analyzed by HPLC, using a Hitachi-Merck LaChrom Elite system with a reverse-phase C18 column (125 × 40 mm ID and 5 µm pore size). The analyses were run, in triplicates, at a flow rate of 1mL/min with a solvent gradient of 1 to 100% of B in A, using 0.05% aqueous TFA as solvent A and MeCN as solvent B, for 30 min, with a 220 nm detection wavelength.

### 4.9. Statistical Analysis

The results regarding the biofilm formation and preformed biofilms were expressed as mean values ± standard deviation (mean ± SD). The statistical significance of differences between controls and experimental groups was evaluated using the Student’s *t*-test. *p*-values of < 0.05 were considered statistically significant.

Results from peptide stability were expressed as the variation of the % of starting compound over time (mean ± SD). Data were analyzed in GraphPad Prism 8.0.1 Software using two-way analysis of variance (ANOVA) followed by Tukey’s multiple comparison test. Statistical significance was considered when *p* < 0.05.

## Figures and Tables

**Table 1:** Minimum inhibitory concentrations (MIC) of MeIm-3.1-PP4 and its parent peptide, 3.1-PP4, against reference (American Type Culture Collection, ATCC) strains and multidrug-resistant (MDR) clinical isolates of Gram-positive and Gram-negative bacteria.

Bacterial Species	Reference Strain or *MDR Isolate*	MIC in μg/mL (in μM)	
		3.1-PP4	MeIm-3.1-PP4
*P. aeruginosa*	ATCC 27853	2.3 (1.2)	1.5 (0.7)
	PA004	1.3 (0.7)	3.1 (1.4)
	Pa3	1.3 (0.7)	1.5 (0.7)
	Pa4	1.3 (0.7)	0.8 (0.4)
*E. coli*	ATCC 25922	1.3 (0.7)	0.8 (0.4)
	Ec2	1.3 (0.7)	1.5 (0.7)
	EC001	1.3 (0.7)	3.1 (1.4)
	EC002	1.3 (0.7)	1.5 (0.7)
	EC003	0.6 (0.3)	1.5 (0.7)
*K. pneumoniae*	KP010	2.3 (1.2)	6.2 (2.9)
	KP004	2.3 (1.2)	49.6 (23.1)
*S. aureus*	ATCC 25923	10.3 (5.3) ^a^	24.8 (11.6) ^a^
	SA007	10.3 (5.3)	12.4 (5.8)
*E. faecalis*	ATCC 29212	41.3 (21.3)	99.3 (46.3)
	Ef1	2.3 (1.2) ^a^	3.1 (1.4)

^a^ Minimum bactericidal concentrations (MBC) were 2× the MIC; in all other cases, MBC equaled the MIC.

## Supplementary Materials

Supplementary Materials can be found at https://www.mdpi.com/1422-0067/21/17/6174/s1. Figures S1–S5, and Tables S1–S2.

## Abbreviations

AMP	Antimicrobial peptide
ATCC	American Type Culture Collection
CFU	Colony-forming units
CLSI	Clinical and Laboratory Standards Institute
CuAAC	Copper(I)-catalyzed azide alkyne cycloaddition
DCM	Dichloromethane
DIEA	*N*-ethyl-*N*,*N*-diisopropylamine
DMF	Dimethylformamide
DMSO	Dimethylsulfoxide
DMSO-d6	Hexadeuterated dimethylsulfoxide
EDTA	Ethylenediaminetetraacetic acid
eq	Molar equivalent
ESI-IT MS	Electrospray ionization—ion trap mass spectrometry
Fmoc	9-fluorenylmethoxycarbonyl
HaCaT	Human epidermal keratinocytes
HBTU	*O*-(benzotriazol-1-yl)-*N,N,N′,N*′-tetramethyluronium hexafluorophosphate
HPLC	High performance liquid chromatography
IL	Ionic Liquid
MBC	Minimum bactericidal concentration
MDR	Multidrug-resistant
MeCN	Acetonitrile
MeIm	1-methylimidazole
MHB II	Cation-adjusted Mueller-Hinton broth
MIC	Minimum inhibitory concentration
MTT	3-(4,5-dimethylthiazol-2-yl)-2,5-diphenyltetrazolium bromide
NMR	Nuclear magnetic resonance
OD	Optical density
PBS	Phosphate buffered saline
Pr-MeIm	Propargyl-1-methylimidazole
RP-HPLC	Reverse-phase high performance liquid chromatography
r.t.	Room temperature
SD	Standard deviation
TFA	Trifluoroacetic acid
TIS	Triisopropylsilane
TMS	Tetramethylsilane
TSB	Tryptic soy broth
