# Supplementary material for: “Clicking” an Ionic Liquid to a Potent Antimicrobial Peptide: On the Route towards Improved Stability
Source: Int J Mol Sci. 2020 Aug 26;21(17):6174. doi: 10.3390/ijms21176174 (PMC7504088; doi:10.3390/ijms21176174)
Supplement: Supplementary file 1 [file ijms-21-06174-s001.pdf]

Supplementary Materials

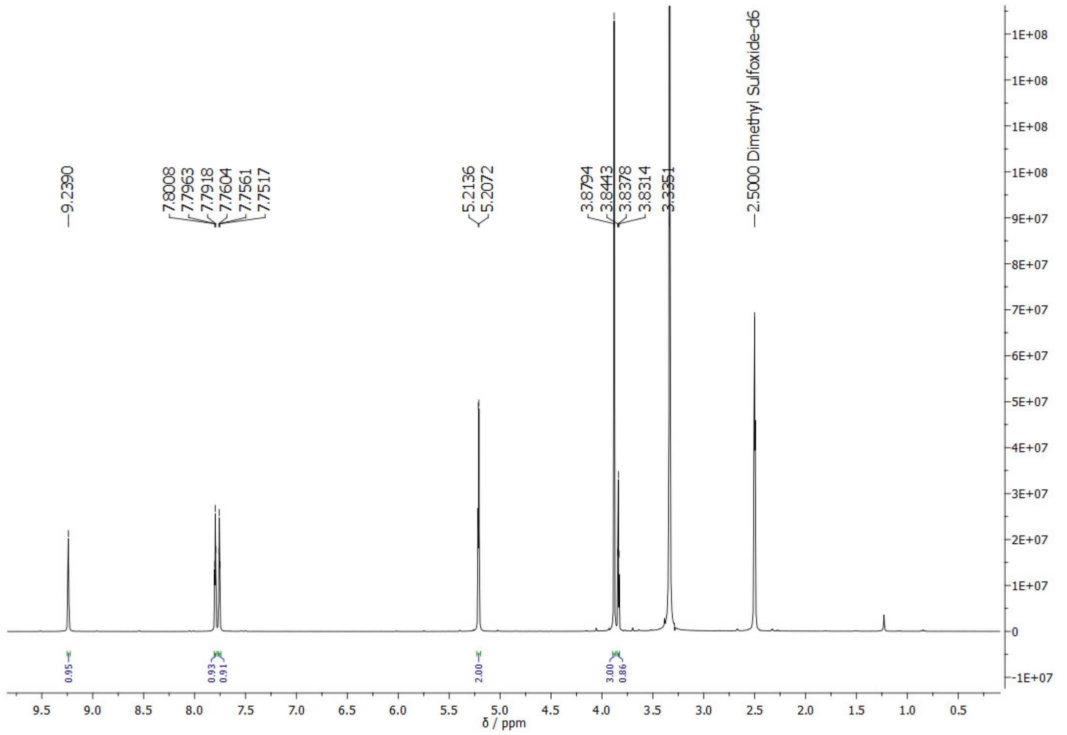

Figure S1. ^{1}H-NMR spectrum of 1-methyl-3-propargyl imidazolium bromide (400 MHz, DMSO-d_6).

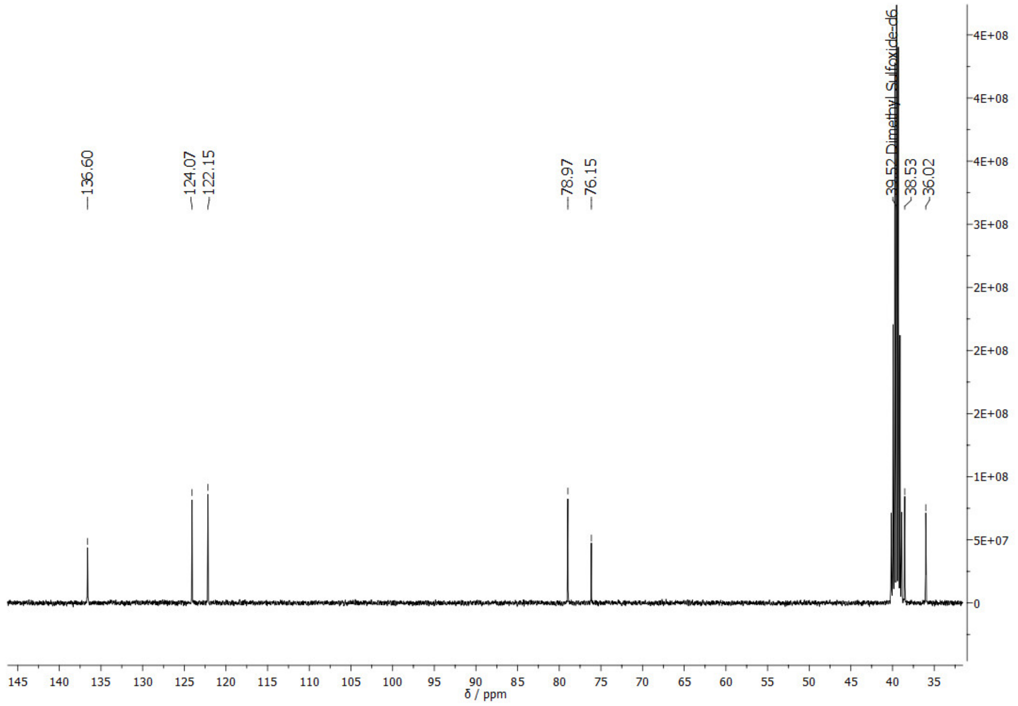

Figure S2. ^{13}C-NMR spectrum of 1-methyl-3-propargyl imidazolium bromide (100 MHz, DMSO-d_6).

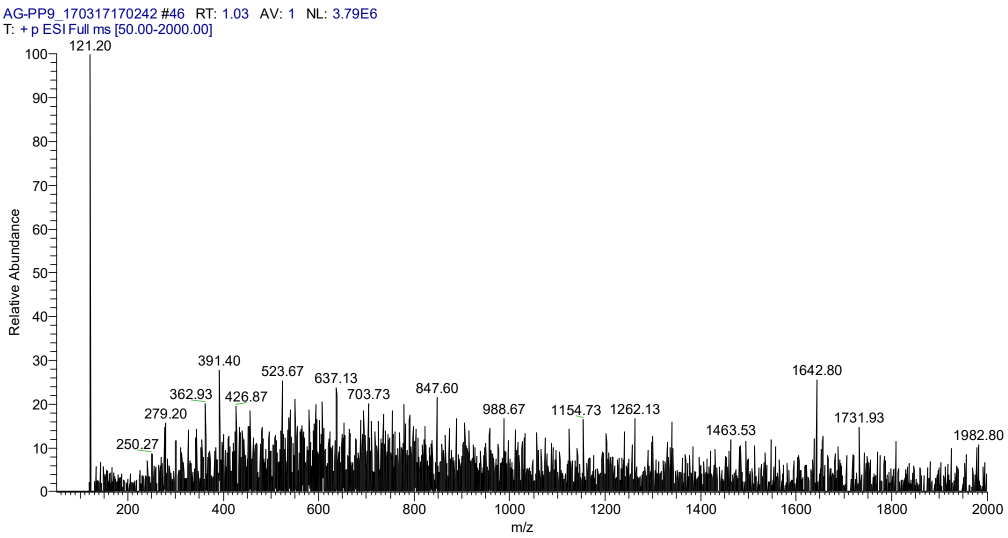

Figure S3. ESI-IT(+) mass spectrum of 1-methyl-3-propargyl imidazolium bromide.

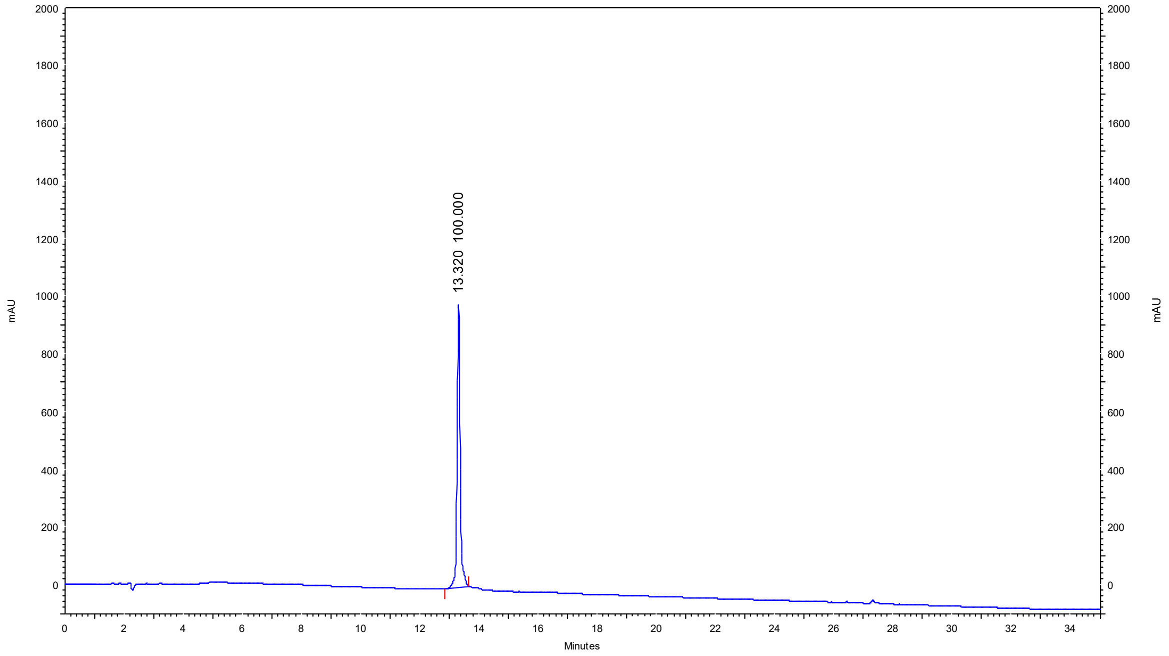

Figure S4. RP-HPLC chromatogram of MeIm-3.1-PP4.

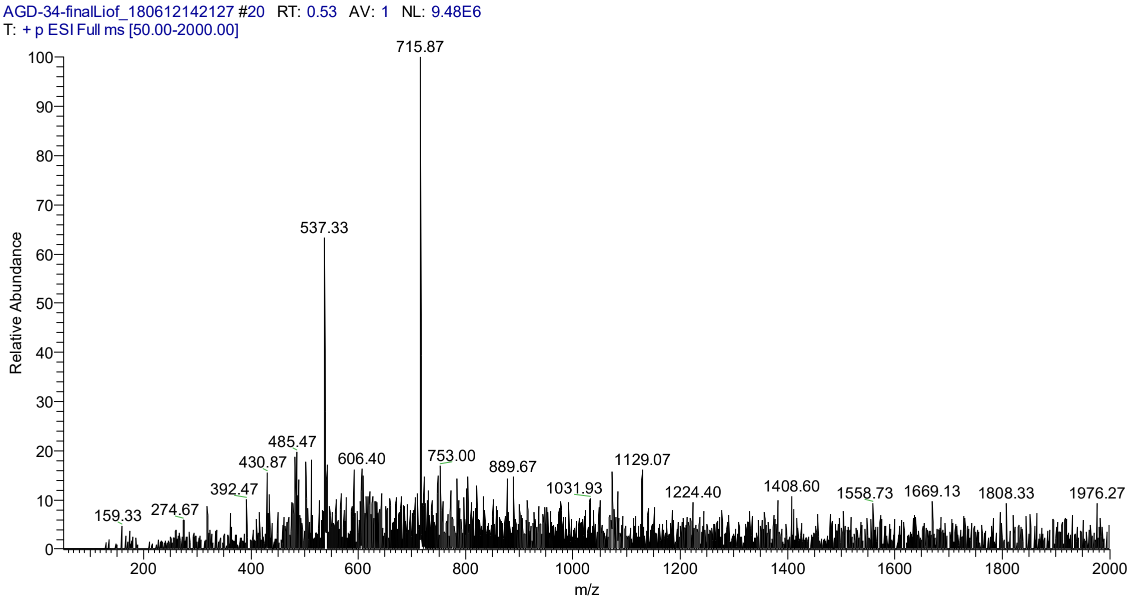

Figure S5. ESI-IT(+) mass spectrum of MeIm-3.1-PP4, where both the tri- and tetra-protonated adducts are observed at m/z 715.87 and 537.33, respectively.

Table 1. Antimicrobial resistance pattern of MDR isolates used in this work.

Isolate	Antimicrobial resistance pattern
PA004	CIP, GEN, IPM, TOB, TZP
Pa3	ATM, CIP, FEP, GEN
Pa4	ATM, CAZ, CIP, FEP, IPM
Ec2	AMP, ATM, CAZ, CIP, CTX, TET
EC001	AMP, CIP, CXM, SXT, LEV
EC002	AMC, AMP, CIP, CXT, CXM, GEN, LEV, SXT, TOB, TZP
EC003	CIP, CXM, LEV
KP004	AMC, AMP, CAZ, CTX, CXM, ERT, MER, SXT, TZP
KP010	AMC, AMP, CAZ, CIP, CTX, CXM, ERT, IPM, LEV, NIT, TZP
Sa007	CIP, CLI, ERI, FOX, GEN, LEV, MOX, OXA
Ef1	AMP, CIP, VAN

AMC: amoxicillin/clavulanic acid; AMK: amikacin; AMP: ampicillin; ATM: aztreonam; CAZ: ceftazidime; CIP: ciprofloxacin; CLI; clindamycin COL: colistin; CTX: cefotaxime; CXM: cefuroxime sodium; ERI: erythromycin; ERT: ertapenem; FEP: cefepime; FOX: cefoxitin GEN: gentamicin; IPM: imipenem; LEV: levofloxacin; MER: meropenem; MOX: moxifloxacin NIT: nitrofurantoin; ; OXA: oxacillin SXT: Trimethoprim/Sulfamethoxazole; TET: tetracycline; TOB: tobramycin; TZP: piperacillin/Tazobactam; VAN: vancomycin

Table S2. Effect of MeIm-3.1-PP4 and 3.1-PP4 at 20×MIC on 24 h preformed biofilms of KP010 (a MDR clinical isolate of *K. pneumoniae*).

Peptide	OD_{600} of planktonic phase of untreated biofilms	OD_{600} of planktonic phase of peptide-treated biofilms	% of Reduction in biofilm proliferation[a]
**3.1-PP4**	0.740 ± 0.047	0.384 ± 0.049	51.8
**MeIm-3.1-PP4**		0.497± 0.050	67.1

[a] *Results are the mean of three independent experiments preformed in triplicate.*
